# Aortic Resection and Replacement for Coral Reef Aorta Involving Severe Obstructive Calcification: A Case Report

**DOI:** 10.7759/cureus.86430

**Published:** 2025-06-20

**Authors:** Yoshiaki Sone, Shunta Hayakawa, Yukihide Numata, Shinji Kamiya, Hideki Sasaki

**Affiliations:** 1 Cardiovascular Surgery, Nagoya City University East Medical Center, Nagoya, JPN

**Keywords:** aortic calcifications, aortic obstruction, aortic stenosis, coral reef aorta, intermittent claudication, open surgery, protruding calcifications

## Abstract

Coral reef aorta (CRA) is a rare aortic condition characterized by calcifications protruding into the lumen, resulting in severe stenosis that can lead to symptoms such as intermittent claudication in the lower limbs and intestinal ischemia. Some patients may progress to fatal conditions such as intestinal ischemia or congestive heart failure. A 74-year-old female was referred to our hospital with intermittent claudication and severe calcification of the abdominal aorta. Computed tomography (CT) scans revealed extensive calcification of the infrarenal abdominal aorta, with protrusions into the lumen, resulting in severe stenosis. After a thorough discussion with catheter interventionists, we concluded that graft replacement was a reasonable option, offering a lower risk of re-intervention compared with endovascular surgery. Considering the sites for aortotomy and anastomosis, we assessed that prosthetic graft replacement was more suitable than endarterectomy or bypass. Under general anesthesia, the patient underwent a laparotomy through a midline abdominal incision. The calcified aorta was resected and reconstructed with a prosthetic graft. She showed improvement in intermittent claudication postoperatively, and she was discharged home on postoperative day 13. No recurrence of symptoms has been observed during the one-year follow-up period.

## Introduction

Coral reef aorta (CRA) is a rare aortic condition characterized by calcifications protruding into the lumen, resulting in severe stenosis. These distinctive calcifications differ markedly from those seen in other vascular calcification disorders in both their morphology and pattern of growth. Qvarfordt et al. [1] first introduced this term in 1984 in their report on nine patients with severe aortic stenotic lesions caused by suprarenal aortic calcification, emphasizing its distinctive presentation. CRA is more common in women, and surgical intervention is performed at an average age of around 60 years [2]. The etiology, pathology, and histological findings of the calcification remain unclear. The pathophysiology is characterized by severe stenosis caused by calcification, leading to distal hypoperfusion and proximal hyperperfusion. In CRA, stenosis of the suprarenal, juxtarenal, or infrarenal aorta can cause symptoms such as intermittent claudication in the lower limbs and intestinal ischemia. Some patients may progress to fatal conditions such as intestinal ischemia or congestive heart failure. Although there is no clear consensus regarding the surgical indications, we consider surgical intervention in symptomatic cases. Endovascular treatment for CRA may result in residual lesions or recurrence due to severe calcification. Open surgery (e.g., thromboendarterectomy or bypass grafting) remains widely regarded as the definitive treatment, even as the progress and growing use of catheter interventions have led to an increase in reports of endovascular therapy for CRA in recent years [2,3].

We report a case of a patient with CRA involving extensive calcification of the infrarenal aorta, where prosthetic graft replacement was performed as the definitive treatment rather than thromboendarterectomy or bypass grafting. In this case, although extensive calcification was observed, a segment of the infrarenal aorta with only mild calcification and suitable for clamping was identified, and the risk associated with open surgery was considered relatively low.

## Case presentation

**Figure 1:**
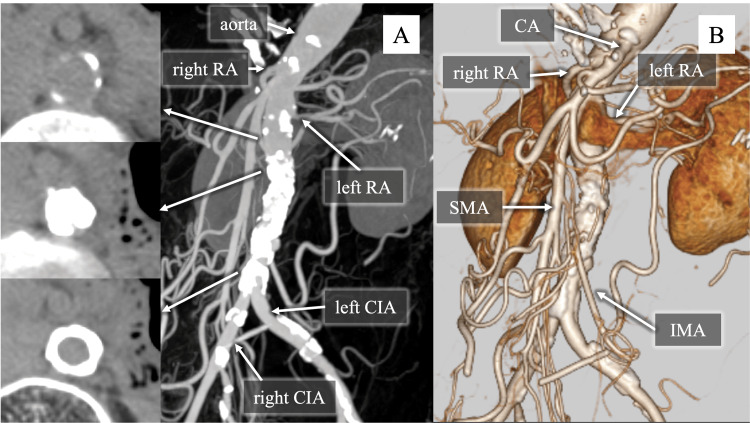
Preoperative CT images (A) The plain CT image reveals extensive calcification of the infrarenal aorta and localized stenotic lesions, whereas only mild calcification is noted in the aorta just below left RA bifurcation and in both common iliac arteries. (B) The contrast-enhanced CT image shows that the CA, SMA, IMA, and both right and left renal arteries are patent, with no significant stenosis. CT: computed tomography, RA: renal artery, CIA: common iliac artery, CA: celiac artery, SMA: superior mesenteric artery, IMA: inferior mesenteric artery

A 74-year-old female was referred to our hospital with intermittent claudication. She had presented to her previous physician one and a half years earlier with intermittent claudication after walking 300 meters and was managed conservatively. At this time, she experienced intermittent claudication after walking only 50 meters, indicating progression of her symptoms. Computed tomography (CT) scans revealed severe calcification of the abdominal aorta. Therefore, she was referred to our hospital for further treatment. Her medical history included hypertension, dyslipidemia, osteoporosis, lumbar compression fractures, and a history of smoking. She was receiving amlodipine and atorvastatin, without any antithrombotic medication. Her height was 1.50 m, her weight was 30.5 kg, and her body mass index was 13.6 kg/m². The bilateral femoral, popliteal, and dorsalis pedis arteries were weakly palpable, but no discoloration of the lower extremities was observed. The laboratory examination indicated no clinically significant abnormal findings relevant to perioperative management, including mild hyponatremia and slightly elevated HbA1c (Table 1). The ankle-brachial index was 0.73 on the right and 0.70 on the left. CT scans showed extensive calcification of the infrarenal abdominal aorta with protrusions into the lumen, resulting in severe stenosis that was nearly occlusive, and localized mild calcification in the segment of the aorta immediately distal to the renal artery bifurcation (Figure 1A). Abdominal branches, including the celiac artery, superior mesenteric artery, and right and left renal arteries, were well enhanced on CT (Figure 1B). A protruding calcification was located between the superior and inferior mesenteric arteries, and the arc of Riolan was well developed. Neither aortic dissection nor extrinsic compression was observed. Since the diagnosis of abdominal aortic stenosis had already been established at the previous hospital, imaging of the lower limbs was not performed. No aortic occlusion was observed, ruling out Leriche syndrome and leading to a diagnosis of CRA. 

**Table 1:** Laboratory examination results

Parameter, unit	Preoperative value	Reference range
White blood cell count, × 10³ /µL	7.3	3.3-8.6
Hemoglobin, g/dL	12	11.6-14.8
Platelet count, × 10³ /µL	210	158-348
Aspartate aminotransferase, U/L	24	13-30
Alanine aminotransferase, U/L	19	7-23
Creatine phosphokinase, U/L	157	41-153
Low-density lipoprotein cholesterol, mg/dL	108	65-163
Creatinine, mg/dL	0.56	0.46-0.79
Sodium, mEq/L	133	138-145
Potassium, mEq/L	4.8	3.6-4.8
Calcium, mEq/L	9.1	8.8-10.1
Hemoglobin A1c, %	6.1	4.9-6.0

**Figure 2:**
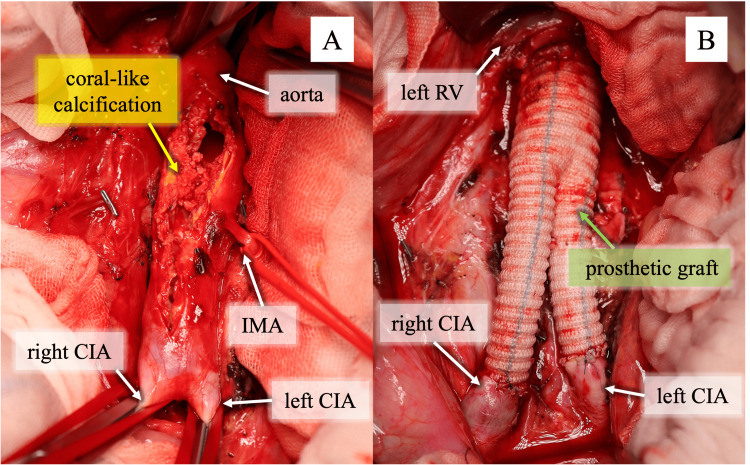
Intraoperative findings (A) An aortotomy was performed after clamping the aorta just below left renal artery bifurcation and both common iliac arteries, revealing localized coral-like calcification and extensive calcification from this site to the aortic bifurcation. (B) The aorta was reconstructed with a J-graft (16 × 8 mm) prosthetic graft. CIA: common iliac artery, IMA: inferior mesenteric artery, RV: renal vein

After the diagnosis and a thorough discussion between cardiovascular surgeons and interventional radiologists, a decision was made to perform CRA resection and revascularization using a prosthetic graft. In open surgery, clamping the aorta just distal to the renal artery bifurcation was considered to carry a low risk of aortic injury or embolism. Prosthetic graft replacement was selected in preference to thromboendarterectomy, considering the risks of reintervention. Laparotomy through a midline abdominal incision was performed on the patient under general anesthesia. The mesenteric vasculature was well developed, forming good collateral circulation. The small intestine was mobilized in a cranial and rightward direction. After systemic heparinization, the aorta just distal to the renal artery bifurcation and both common iliac arteries were clamped. No perfusion to the lower extremities was performed during aortic clamping. As there was no suitable site for creating a bypass anastomosis between the aortic clamp site and the calcified segment, prosthetic graft replacement was selected. The calcified aorta was resected and reconstructed with a 16 × 8 mm J-graft (Japan Lifeline, Tokyo, Japan) (Figure 2). The inferior mesenteric artery was ligated at its origin because of its small caliber. The total operation time was 212 minutes, including 46 minutes of ischemia in the right lower limb and 60 minutes in the left.

**Figure 3:**
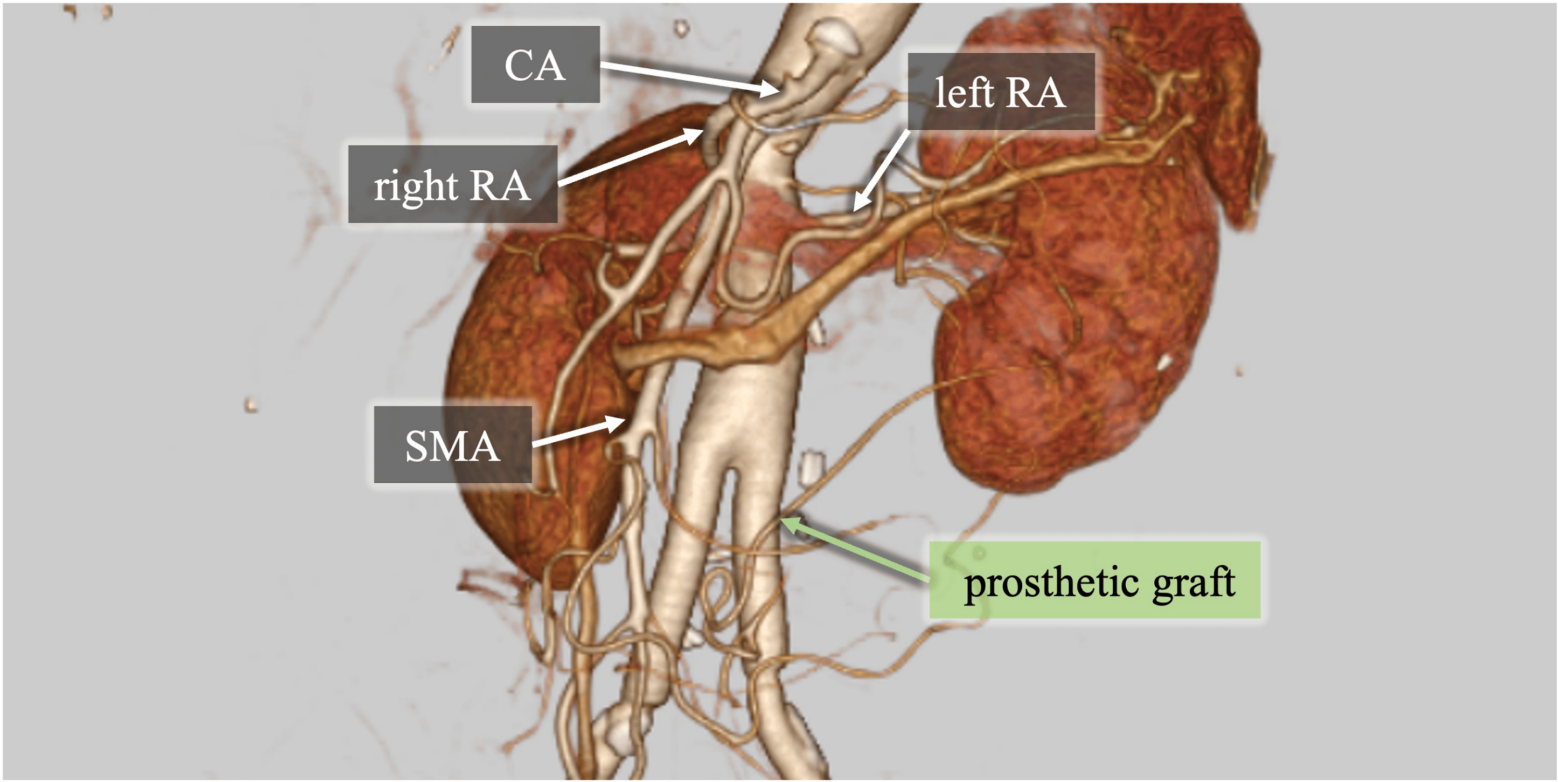
Postoperative CT imaging All anastomotic sites showed no significant stenosis, and the arteries to both lower limbs remained patent. The CA, SMA, and both right and left renal arteries were consistent with their preoperative condition. CT: computed tomography, RA: renal artery, CA: celiac artery, SMA: superior mesenteric artery

After extubation in the operating room, the patient was transferred to the intensive care unit and remained there for 18 hours. After surgery, her intermittent claudication resolved, and the pulses in the bilateral femoral, popliteal, and dorsalis pedis arteries were markedly enhanced. Atrial fibrillation was observed on postoperative day three, and bisoprolol and edoxaban were initiated. No postoperative deterioration in renal function or anemia was observed. The ankle-brachial index improved to 1.05 on the right and 1.03 on the left. Enhanced CT imaging performed on postoperative day eight revealed good patency in both the right and left limbs of the graft, with no impairment of the abdominal branches (Figure 3). She was discharged home on postoperative day 13. After discharge, non-contrast CT has been used for imaging follow-up at six months and a year after surgery, followed by annual evaluations thereafter. A year after surgery, the patient remained symptom-free, and follow-up CT revealed no anastomotic pseudoaneurysm.

## Discussion

Although the etiology and pathogenesis of CRA remain unknown, its development may be associated with an initial injury to the aortic endothelium. According to Minnee et al. [3], repetitive contractions of the crux of the diaphragm cause localized injury to the suprarenal abdominal aorta, which is speculated to contribute to the development of CRA. In addition, the serum calcium level is usually within the normal range. In our patient, the pathological lesion was located in the infrarenal aorta, and the exact cause remained unclear. Moreover, her serum calcium level was within the normal range. 

The clinical symptoms of CRA, which are determined by the location and extent of calcification, include intermittent claudication, refractory hypertension, intestinal angina, weight loss, renal insufficiency, and rest pain [2]. Some patients may progress to fatal conditions, such as intestinal necrosis caused by ischemia or congestive heart failure due to afterload mismatch [3,4,5]. Intermittent claudication and other symptoms may also be present in lower extremity artery disease. It is important to perform CT with consideration of CRA in the differential diagnosis, as the characteristic calcifications in the aorta are not observed in lower extremity artery disease. In our patient, CT of the lower extremities was not performed, since CRA had already been diagnosed at the previous hospital. She presented with progressive intermittent claudication. Although no consensus exists regarding the optimal timing of surgery for CRA, surgical intervention was considered appropriate in this case because the patient's symptoms worsened despite medical treatment at the previous hospital. 

In terms of aortic stenotic or obstructive diseases, Leriche syndrome is one of the pathologies similar to CRA. Commonly referred to as an aortoiliac occlusive disease, Leriche syndrome is characterized by the classic triad of erectile dysfunction, intermittent claudication, and the absence of lower extremity pulses. It is typically a chronic complete aortic occlusive disease attributed to arteriosclerosis, whereas CRA is a severe stenotic lesion caused by calcification. Given that CRA results in diminished distal perfusion beyond the stenotic segment, it can produce similar clinical features, including the triad. Leriche syndrome frequently results from retrograde thrombotic occlusion. According to Ligush et al. [6], the pathogenesis of infrarenal aortic occlusion involves the progression of iliac and distal aortic atherosclerosis, leading to infrarenal aortic thrombosis. Atherosclerosis is a chronic pathophysiological process characterized by endothelial injury, lipid accumulation, and immune cell infiltration leading to plaque formation within the arterial wall. Plaque rupture exposes thrombogenic material, triggering platelet activation and thrombus formation that may acutely occlude arteries such as the aorta, causing ischemic events. On the other hand, vascular calcification in the aorta is an active, cell-mediated process resembling bone formation, rather than a passive accumulation of calcium [7]. The vascular expression of pro-osteogenic morphogens is regulated by physiological stimuli that promote calcification, such as inflammation, shear stress, oxidative stress, hyperphosphatemia, and elastinolysis. These stimuli not only drive calcification but also compromise defense mechanisms that limit calcium deposition in the vasculature. In our patient, almost complete obstruction due to severe calcific lesions was evident, but no obvious thrombus was observed on preoperative CT or intraoperative findings. Therefore, she had not progressed to Leriche syndrome.

In recent years, surgeons have reported various endovascular treatments for CRA, including stent placement, balloon angioplasty, or lithotripsy [8-11]. Chag et al. [11] performed endovascular treatment using intravascular lithotripsy for a case of juxtarenal CRA, with no complications, and the patient remained free of symptom recurrence at six months postoperatively. Compared with open surgery, endovascular therapy is less invasive and associated with fewer iatrogenic perioperative complications and has demonstrated comparable short-term outcomes in symptom improvement [2]. However, the long-term outcomes of endovascular treatment for CRA remain unclear, and these treatments may not be suitable when the lesion involves critical branches or when the calcification is particularly advanced. Conversely, although open surgery, such as graft replacement, is more invasive and carries a higher risk of mortality, it remains a traditional approach that can offer a more definitive treatment option with better long-term patency [9]. Open surgery is preferred for long-term durability, while endovascular options may be appropriate in selected patients with comorbidities or compromised general status. For our patient, we opted for open surgery as a relatively low-risk definitive treatment option after considering the extensive calcification (which posed high risks of restenosis and re-intervention in endovascular therapy such as stent expansion or lithotripsy) and the localization of the lesion to the infrarenal abdominal aorta, which allowed us to avoid intervention in the suprarenal aorta. Although intravascular lithotripsy is a promising alternative with a low incidence of embolic complications, Residual lesions may lead to restenosis. In addition, the absence of calcification in the aorta below the renal artery bifurcation and the bilateral common iliac arteries facilitated safe clamping, reducing the risk of clamping-site injury and abdominal ischemia.

Baldaia et al. [2] reviewed open surgery performed on 110 (88.7%) of 124 patients with CRA, with the following methods applied: endarterectomy alone in 87 patients (79.1%), extra-anatomic bypass alone in eight patients (7.3%), endarterectomy combined with extra-anatomic bypass in seven patients (6.4%), and aortic graft replacement in a patient (0.9%). Among patients who underwent open surgery, the in-hospital mortality rate was 10.9%, the morbidity rate was 28.2%, and the long-term all-cause mortality at 180 months of follow-up was 30%. These high rates may be related to the frequent presence of thoracoabdominal lesions, which required highly invasive thoracoabdominal approaches in more than half of the cases, as well as the patients’ cardiovascular burden and comorbidities. All 17 patients who required re-intervention had undergone thromboendarterectomy alone, accounting for 20% of this treatment group. In our patient, the severely calcified lesion was confined to the infrarenal to aortic bifurcation. After a thorough discussion with catheter interventionists, we concluded that open surgery was a reasonable option, offering a lower risk of re-intervention compared with endovascular surgery. Although an aorto-iliac bypass can be an option in selected cases, it was not suitable in ours. Due to severe calcification extending from the infrarenal aorta to the common iliac arteries, there was no safe space to place a side clamp around the infrarenal aorta, which would have increased the risk of embolism. Considering the feasibility, long-term graft patency, and lower risk of embolic complications, we determined that graft replacement of the entire calcified segment - from the infrarenal aorta to both common iliac arteries - was a more appropriate approach in our case. Graft replacement was considered preferable to endarterectomy because the former can be performed relatively easily for infrarenal aortic lesions, whereas the latter carries a risk of re-intervention due to postoperative restenosis. We believe that our technique, which involves near-complete exclusion of the calcified segment and replacement with a prosthetic graft, offers the potential for reduced morbidity and a lower re-intervention rate in selected patients, as demonstrated in our case.

## Conclusions

Although CRA is a rare condition, it is important to recognize that it can occasionally result in life-threatening complications such as intestinal ischemia or congestive heart failure. Appropriate treatment can result in improvement of both the condition and symptoms. Endovascular treatment has become more common in recent years, but its long-term outcomes remain unclear. Surgical treatment is invasive and associated with high rates of mortality and morbidity. Thromboendarterectomy carries a high risk of reintervention. However, bypass surgery and graft replacement can offer better long-term patency, emphasizing the importance of selecting the most appropriate surgical approach for each individual case. In this case, the patient suffered from intermittent claudication and underwent surgery to alleviate the symptoms and improve their condition. Given that the calcified lesion was limited to the infrarenal aorta and surgical invasiveness was considered relatively low, graft replacement with favorable long-term patency was selected. She showed improvement in intermittent claudication postoperatively and has had no symptoms during the one-year follow-up.
